# Supplementary material for: Pro-phagocytic function and structural basis of GPR84 signaling
Source: Nat Commun. 2023 Sep 14;14:5706. doi: 10.1038/s41467-023-41201-0 (PMC10502086; doi:10.1038/s41467-023-41201-0)
Supplement: Supplementary file 1 — Supplementary Information [file 41467_2023_41201_MOESM1_ESM.pdf]

Supplementary Information

Pro-phagocytic function and structural basis of GPR84 signaling

Xuan Zhang [1, 2, &], Yujing Wang[1, &], Shreyas Supekar[3, &], Xu Cao[4], Laura Jenkins[5], Sara Marsango[5], Jingkai Zhou[4], Jessica Dang[4], Siqi Chen[4], Xiu Li[1], Guibing Liu[1], Graeme Milligan[5, *], Mingye Feng[4, *], Hao Fan[3, *], Weimin Gong[1, *], Cheng Zhang[2, *]

[1]Division of Life Sciences and Medicine, University of Science and Technology of China, Hefei, Anhui, China.

[2]Department of Pharmacology and Chemical Biology, University of Pittsburgh School of Medicine, University of Pittsburgh, Pittsburgh, PA 15261, USA.

[3]Bioinformatics Institute (BII), Agency for Science, Technology and Research (A*STAR), 138671, Singapore.

[4]Department of Immuno-Oncology, Beckman Research Institute, City of Hope Comprehensive Cancer Center, Duarte, CA 91010, USA.

[5]Centre for Translational Pharmacology, School of Biomolecular Sciences, College of Medical, Veterinary and Life Sciences, University of Glasgow, Glasgow G12 8QQ, Scotland, U.K.

[&]Equal contribution

[*]Corresponding authors: G.M. (Graeme.Milligan@glasgow.ac.uk), M.F. (mfeng@coh.org), H.F. (fanh@bii.a-star.edu.sg), W.G. (wgong@ustc.edu.cn), C.Z. (chengzh@pitt.edu).

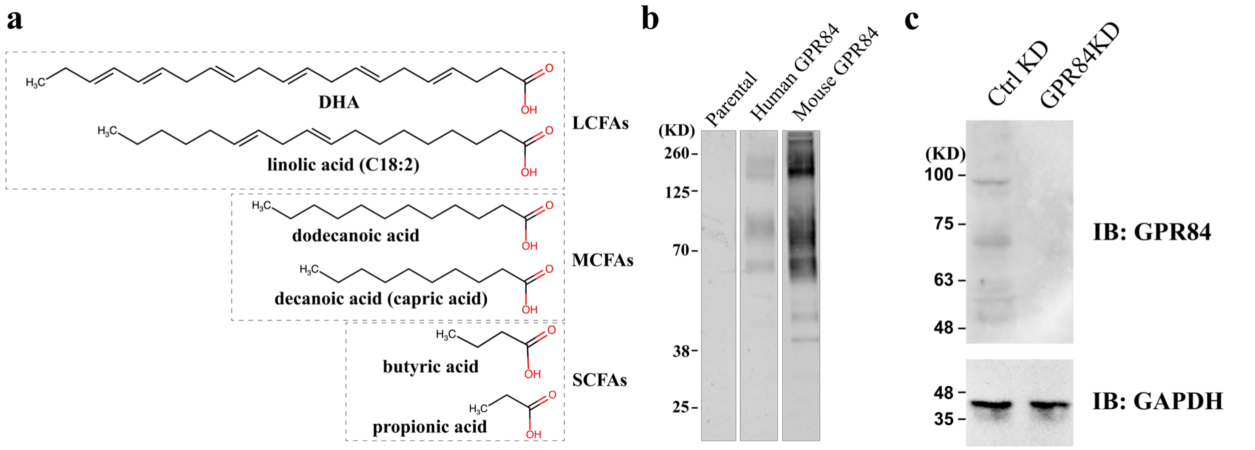

Supplementary Figure 1. (a) Representative short-chain, medium-chain, and long-chain fatty acids (SCFAs, MCFAs, and LCFAs). **(b)** Validation of an in-house anti-GPR84 antiserum by western blot. Flp-In T-REx 293 cells were transfected to express human or mouse GPR84-eYFP. The receptor was then enriched via GFP-trapping and run on SDS-PAGE gels. Immunoblot was performed to validate the in-house GPR84 antiserum raised against mouse receptor. **(c)** Immunoblots with GPR84 antiserum and anti-GAPDH antibody showing knockdown of GPR84 expression in macrophages.

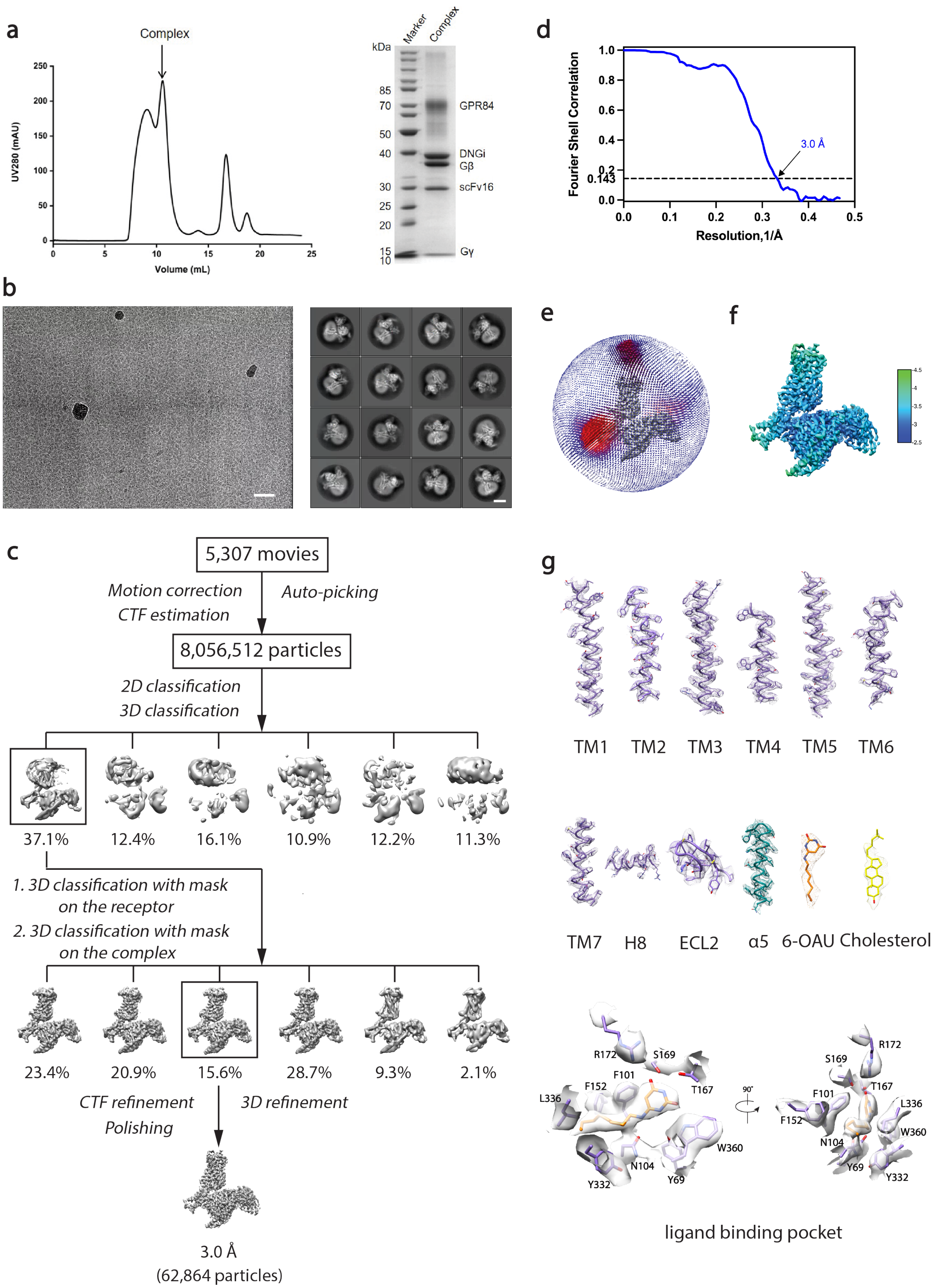

Supplementary Figure 2. Purification of the GPR84-G$_i$ complex with 6-OAU and cryo-EM data processing. (a) Size-exclusion chromatography profile and SDS-PAGE analysis of the purified 6-OAU-GPR84-G$_i$ complex. The data represents results from one-time experiment. **(b)**

Representative cryo-EM micrograph (scale bar: 50 nm) and 2D class averages (scale bar: 5 nm). **(c)** Cryo-EM image processing workflow for the 6-OAU-GPR84-G_i complex. 5307 cryo-EM movies collected on one grid in one session were processed. **(d)** Gold-standard Fourier shell correlation (FSC) curve showing an overall resolution is 3.0 Å at FSC=0.143. **(e)** Angular distribution of the particles used in the final reconstruction. **(f)** Density map according to local resolution estimation. **(g)** Cryo-EM density maps and models of the seven transmembrane helices (TM1-7), Helix 8 (H8), extracellular loop 2 (ECL2), α5 helix of $G\alpha_i$, 6-OAU, cholesterol and ligand binding pocket. The EM density is shown at 0.0445 threshold. 6-OAU is colored in orange.

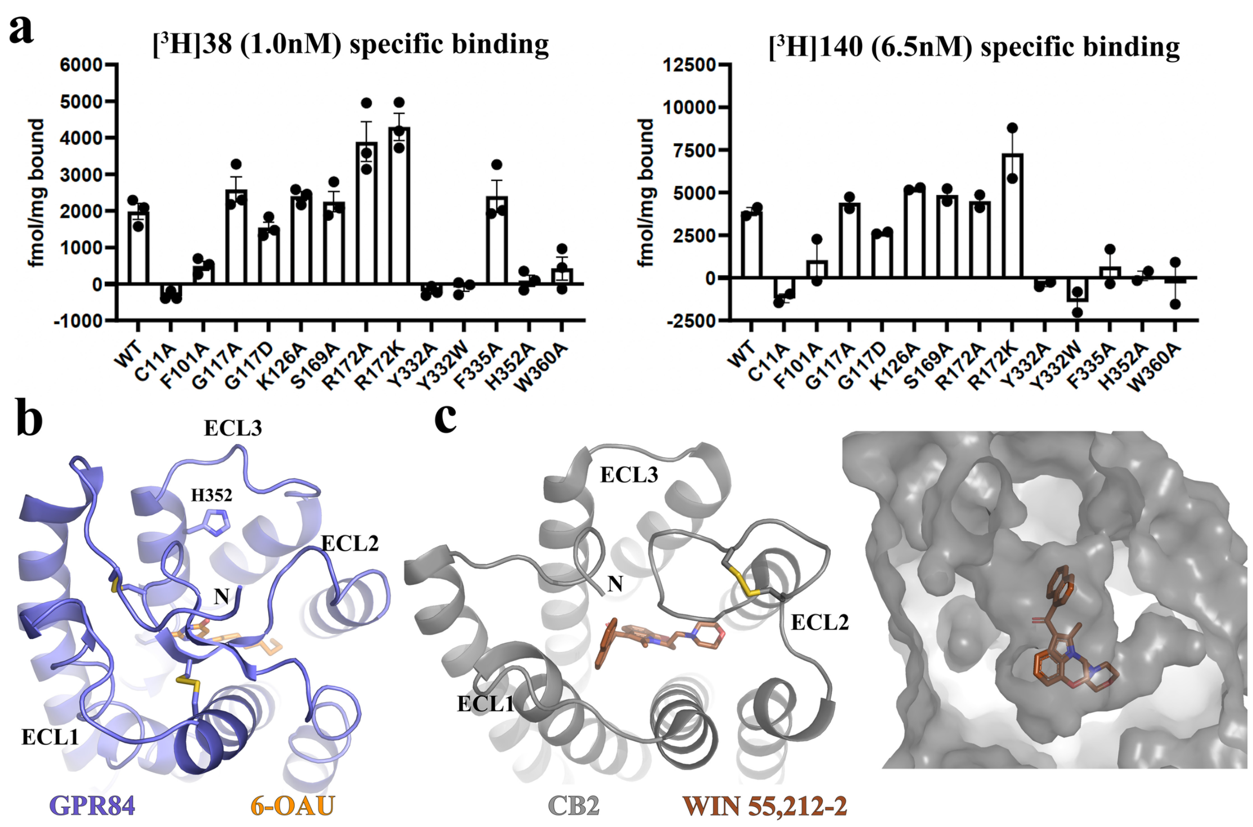

Supplementary Figure 3. Radioligand binding in GPR84 and mutants and structural comparison with CB2. (a) Specific binding of tritiated GPR84 antagonists [^{3}H]38 (1.0 nM) and [^{3}H]140 (6.5 nM) to HEK293T cell membranes transiently transfected to express wild type GPR84 (WT) and the indicated mutants. Each column represents means ± S.E.M. of replicate data from a single experiment that was repeated 3 times with similar outcomes. **(b)** Extracellular region of GPR84. The two disulfide bonds are shown as yellow sticks. 6-OAU is shown as orange sticks. **(c)** Extracellular region of CB2 and occluded ligand binding pocket. WIN 55,212-2 is a CB2 agonist. The figure is based on the CB2 structure with the PDB code 6PT0.

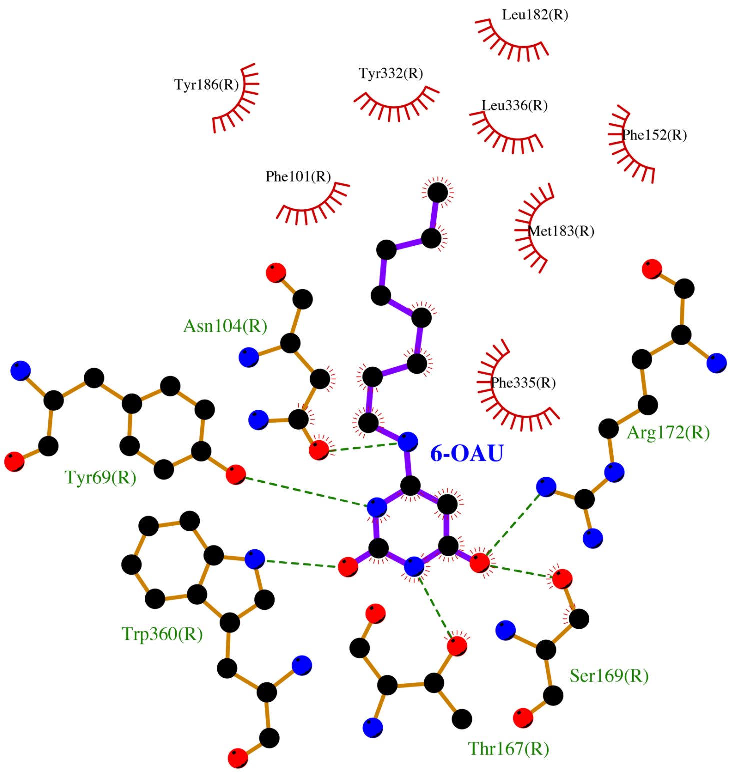

Supplementary Figure 4. Ligplot schematic representation of GPR84 interactions with 6-OAU. The carbon, nitrogen, and oxygen atoms are colored in black, blue, and red, respectively. Residues in GPR84 that form polar and hydrophobic interactions with 6-OAU are shown with green and black labels, respectively. Polar interactions are shown as green dashed lines. The ligand is shown as purple sticks.

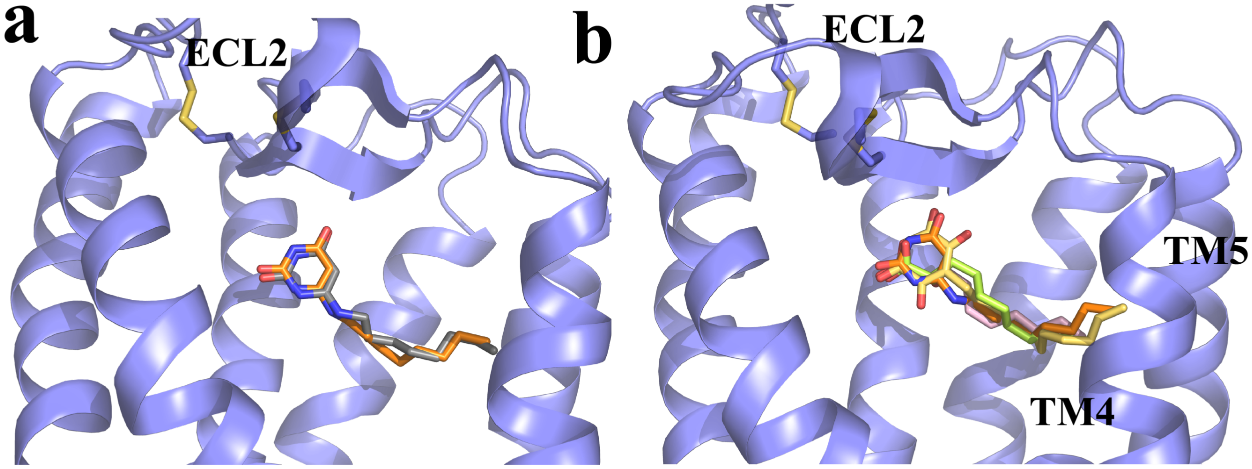

Supplementary Figure 5. Docking of GPR84 agonists. (a) Superimposition of docked 6-OAU (grey) and 6-OAU in our cryo-EM structure (orange). **(b)** Superimposition of docked embelin (light yellow), capric acid (pink), 2-hydroxyl capric acid (lime), and 6-OAU in our cryo-EM structure (orange).

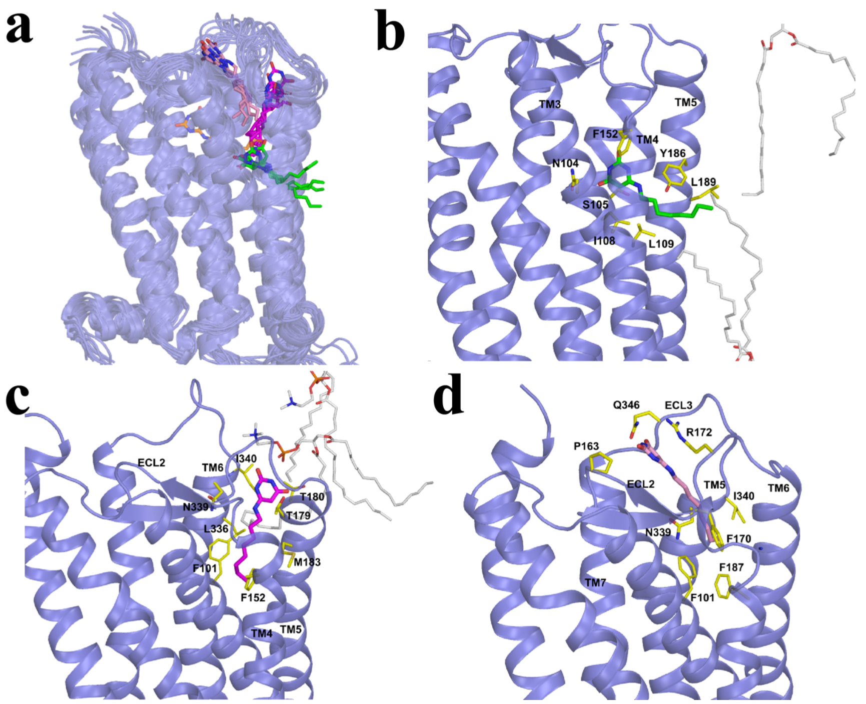

Supplementary Figure 6. Interactions of 6-OAU with GPR84 at three metastable sites. (a) Representative binding poses of 6-OAU at the metastable site S1 (green), S2 (magenta), and S3 (pink). 6-OAU in the cryo-EM structure is shown as orange sticks for reference. **(b)** S1 located at the interface between TM3-TM4-TM5 and membrane lipids where 6-OAU makes hydrophobic contacts with lipid tails and residues I108, L109, Y186, and L189. **(c)** S2 located at the interface between TM5-TM6 and membrane lipids where 6-OAU makes H-bonds with membrane lipid headgroups and hydrophobic contacts with F101, F152, M183, L336, and I340. **(d)** S3 located on top of the orthosteric site near the ECL2-ECL3-water interface where 6-OAU makes cation-π interaction with R172 (presumably acting as a gatekeeper residue) from ECL2, hydrogen bonds with Q346 from ECL3, and hydrophobic contacts with F101, F170, F187, and I340. Lipid molecules from the environment are shown as light grey sticks.

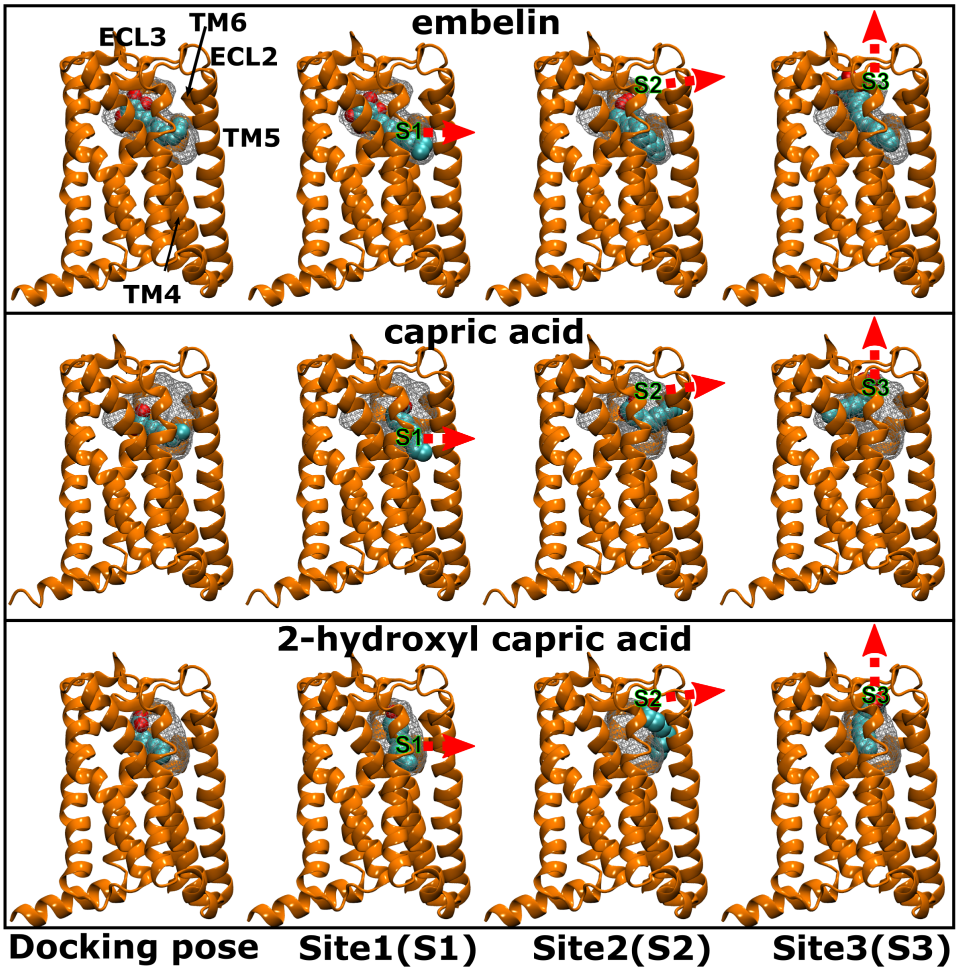

Supplementary Figure 7. Ligand dynamics from MD simulations of embelin, capric acid, and 2-hydroxy capric acid in complex with GPR84. Putative exit routes (metastable sites S1, S2 and S3) for embelin, capric acid, and 2-hydroxy capric acid shown in top, middle and bottom panels, respectively. Ligand densities in the simulations shown in grids. The docked pose and MD-generated conformations captured in the metastable sites S1, S2 and S3 shown for each ligand from left to right, respectively.

a)

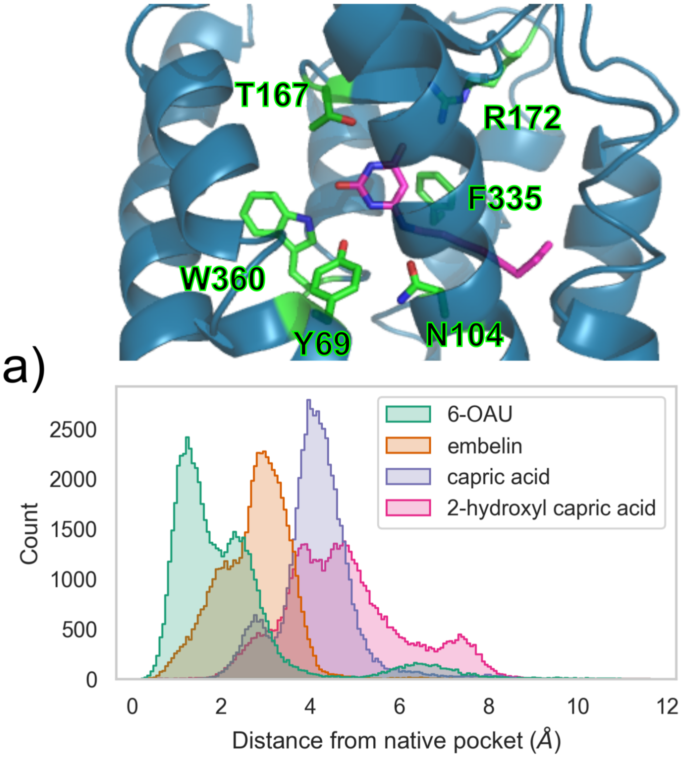

b)

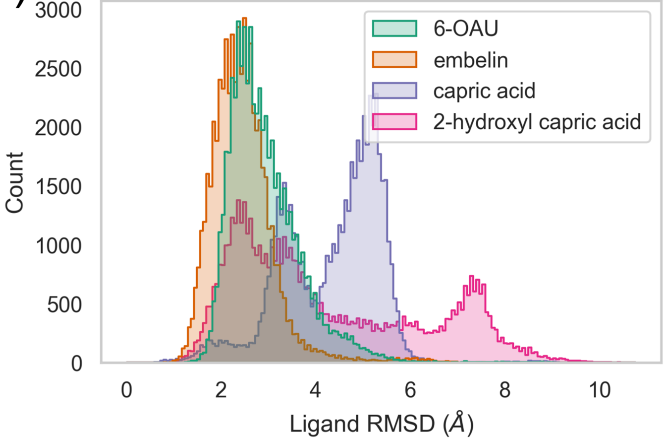

Supplementary Figure 8. Distributions of ligand dynamics from the holo MD simulations. a) Distributions of the distance between center-of-mass of a ligand and the center-of-mass of native site residues shown in green, orange, blue and pink, for 6-OAU, embelin, capric acid, and 2-hydroxy capric acid, respectively. Top: active site residues R172, T167, W360, Y69, N104 and F335 employed for native cavity center-of-mass calculation shown in green, and native ligand (6-OAU) in pink. Bottom: distributions of distance between the active site centroid and the respective ligand center-of-mass. **b)** Distributions of root-mean-squared-deviation (RMSD) of 6-OAU, embelin, capric acid, and 2-hydroxy capric acid during MD simulations shown in green, orange, blue and pink, respectively. The respective docking pose was used as the reference for RMSD calculation for each ligand. Counts in distribution plots represent the number of snapshots captured every 0.1 ns during the 5 µs MD simulations that produced 50000 snapshots for each ligand.

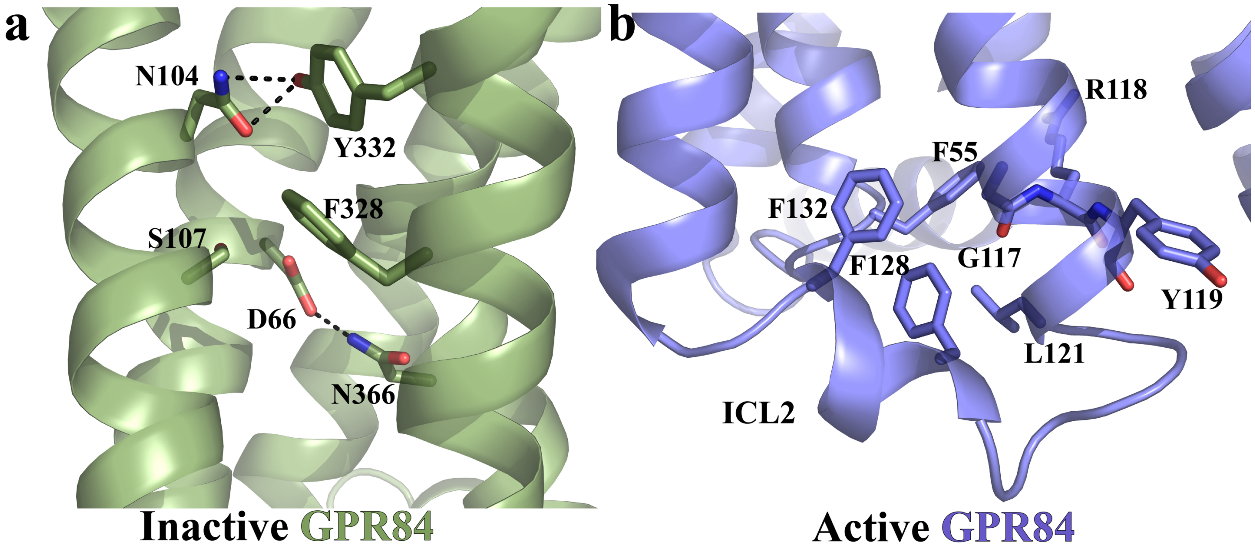

Supplementary Figure 9. (a) Residues at the core region of GPR84 in the Alphafold predicted inactive structure. **(b)** GRY motif and ICL2 in the active GPR84. Polar interactions are shown as dashed lines.

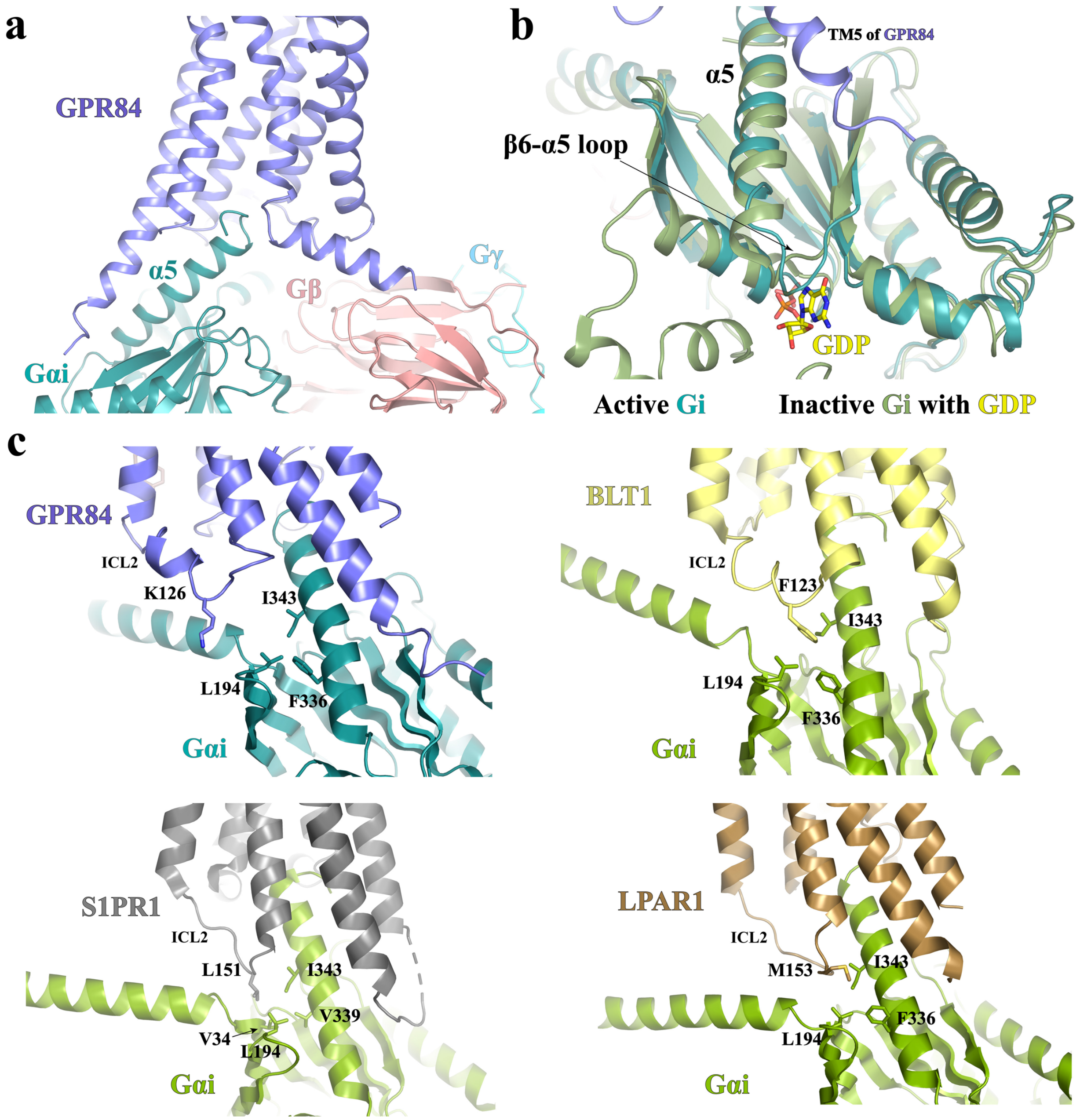

Supplementary Figure 10. Gi coupling and activation. (a) α5 of $G_{\alpha i}$ as the major interaction site for GPR84. GPR84 is colored in blue. Gαi, Gβ and Gγ subunits are colored in cyan, pink and light blue, respectively. **(b)** Conformational changes of α5 and the β6-α5 loop in Gαi during G protein activation and GDP release. The inactive Gαi with GDP is colored in light green. GDP is colored in yellow. **(c)** Comparison of the Gi-coupling to GPR84, BLT1 (PDB ID 7VKT), S1PR1 (PDB ID 7TD3), and LPAR1 (PDB ID 7TD0) at ICL2. In each of these lipid GPCRs except GPR84, a hydrophobic residue in ICL2 engages in hydrophobic interactions with a similar set of residues of Gαi. GPR84, BLT1, S1PR1, and LPAR1 are colored blue, dark yellow, grey, and brown, respectively. Gαi that couples to BLT1, S1PR1, or LPAR1is colored in lime.

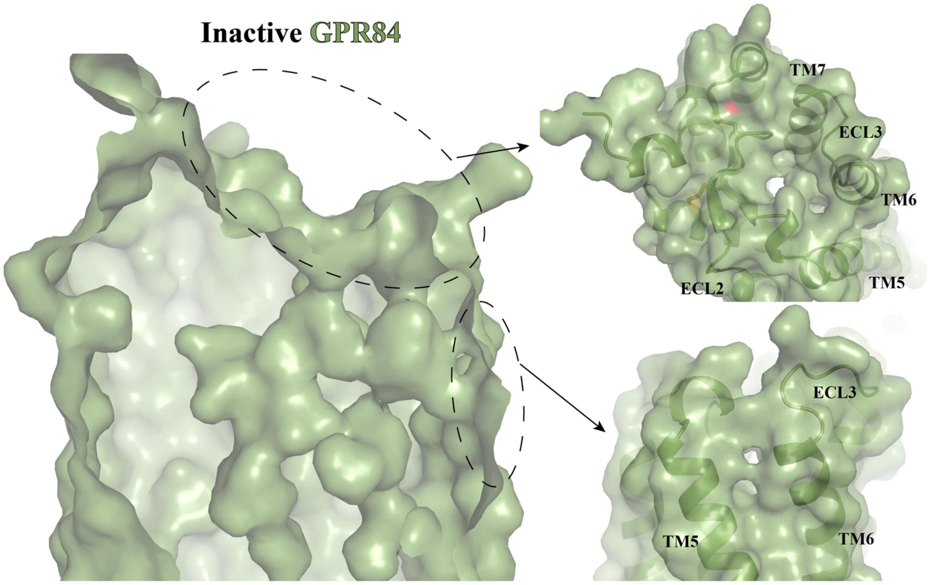

Supplementary Figure 11. Potential ligand binding pocket with openings in the Alphafold predicted inactive structure of GPR84.

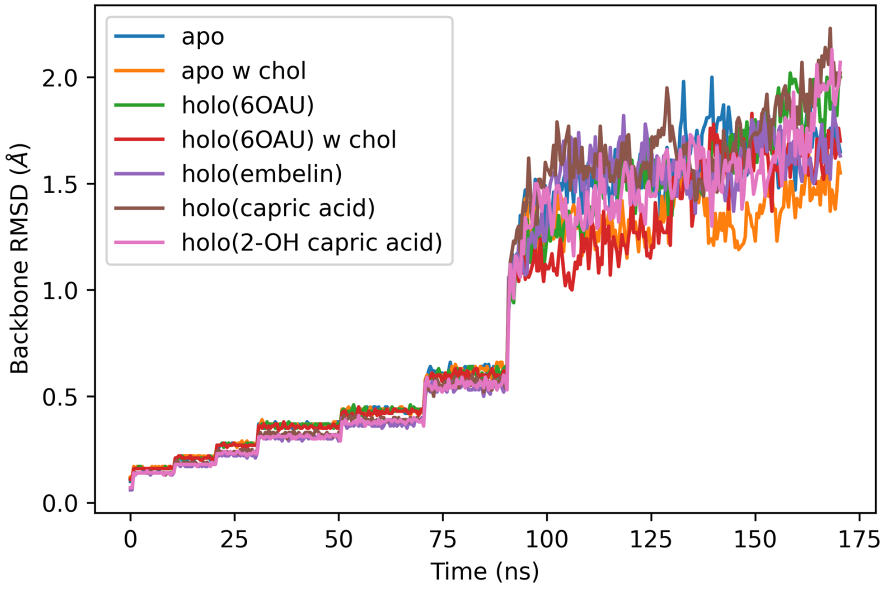

Supplementary Figure 12. Root-mean-squared-deviation (RMSD) of the protein backbone atoms in equilibration phase of GPR84 apo and holo MD simulations with or without cholesterol (chol).

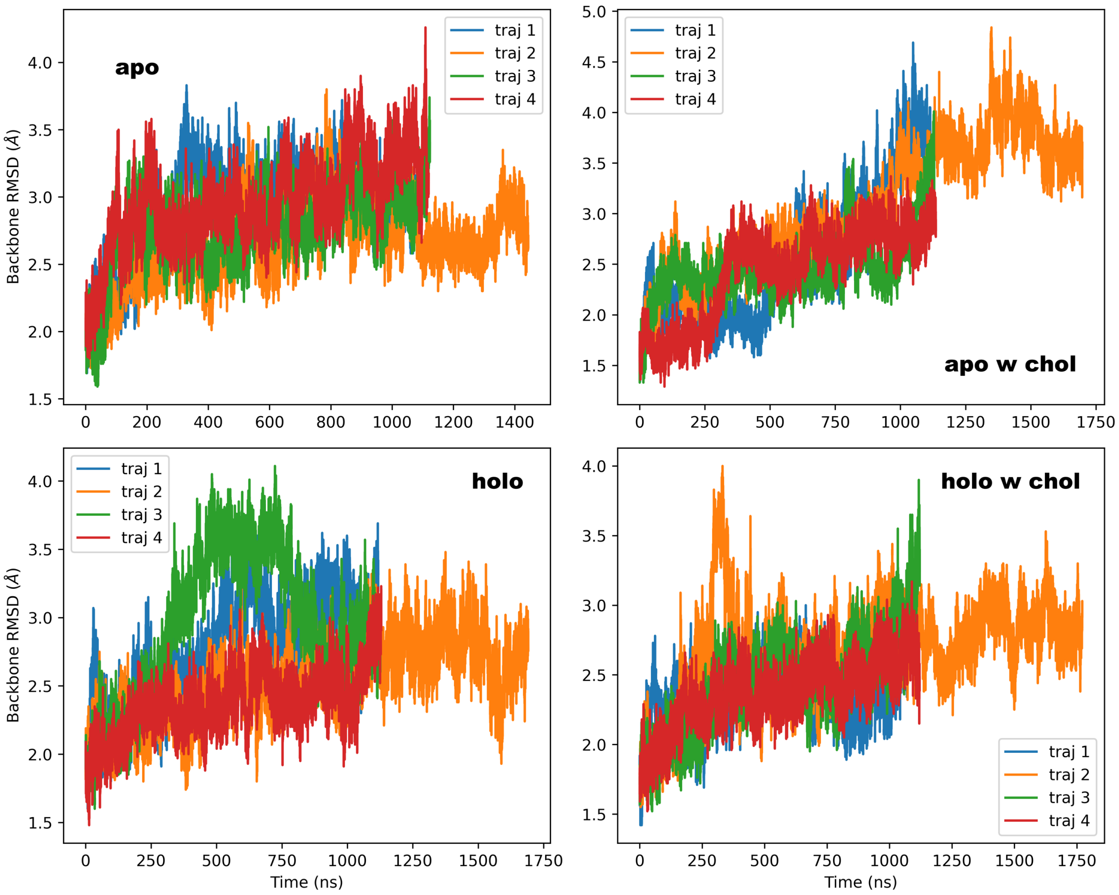

Supplementary Figure 13. Root-mean-squared-deviation (RMSD) of the protein backbone atoms for the production runs of GPR84 apo and holo(6OAU) MD simulations with or without cholesterol (chol).

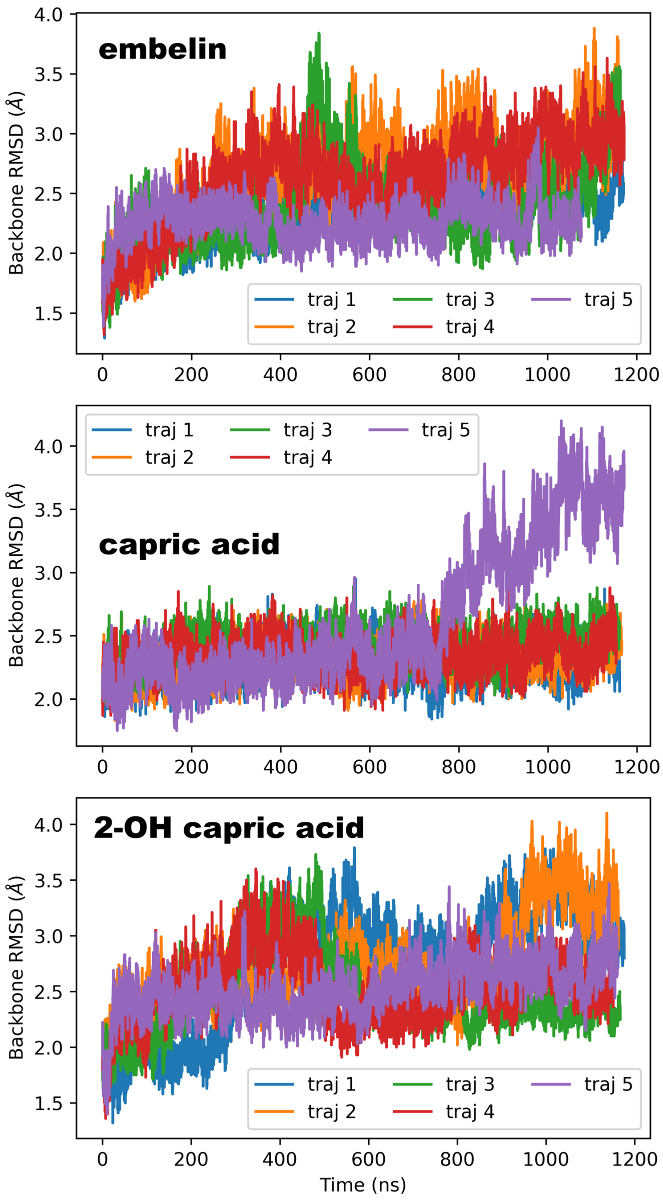

Supplementary Figure 14. Root-mean-squared-deviation (RMSD) of the protein backbone atoms for the production runs of holo MD simulations with embelin, capric acid or 2-hydroxyl capric acids bound to GPR84.

Supplementary Table 1. Cryo-EM data collection and refinement statistics

	6-OAU-GPR84-Gi complex (EMD-29645, PDB 8G05)
Data collection and processing	
Magnification	81,000
Voltage (kV)	300
Electron exposure ($e^-/Å^2$)	55
Defocus range (μm)	-1.2 to -2.2
Pixel size (Å)	1.07
Symmetry imposed	C1
Initial particle images (no.)	8,056,512
Final particle images (no.)	62,864
Map resolution (Å)	3.0
FSC threshold	0.143
Map resolution range (Å)	2.5-4.5
Refinement	
Model resolution (Å)	3.1
FSC threshold	0.5
Model composition	
Non-hydrogen atoms	8955
Protein residues	1142
Ligand	1
Lipids	1
R.m.s. deviations	
Bond lengths (Å)	0.008
Bond angles (°)	0.923
Validation	
MolProbity score	1.45
Clashscore	4.43
Rotamer outliers (%)	0.72
Ramachandran plot	
Favored (%)	96.45
Allowed (%)	3.55
Disallowed (%)	0

Supplementary Table 2. Scores of docking 6-OAU, embelin, capric acid, and 2-hydroxy capric acid to the receptor cryo-EM structure.

Ligand	Docking score
**6-OAU**	-12.52
**Embelin**	-8.76
**Capric acid**	-4.45
**2-hydroxy capric acid**	-5.51

Supplementary Table 3. System setup for MD simulations

**Simulation box dimensions**	X_{span}=78Å; Y_{span}=78 Å; Z_{span}=110 Å
**System size**	*ca.* 75000 atoms
**Lipid composition**	POPC:DOPC::1:1
**Total lipids**	156
**Water molecules**	*ca.* 14900
**Ions**	Na^+/Cl^-
**Ion concentraion**	0.15 M
**Forcefield**	CHARMM36
